# Variable Pacing Is Associated with Performance during the OCC^®^ Ultra-Trail du Mont-Blanc^®^ (2017–2021)

**DOI:** 10.3390/ijerph20043297

**Published:** 2023-02-13

**Authors:** Pedro Corbí-Santamaría, Alba Herrero-Molleda, Juan García-López, Daniel Boullosa, Vicente García-Tormo

**Affiliations:** Faculty of Physical Activity and Sports Sciences, AMRED, Human Movement and Sports Performance Analysis, Universidad de León, 24071 León, Spain

**Keywords:** trail running, pace variability, sex difference, endurance exercise

## Abstract

The current evidence suggests that pacing may not be affected by performance level or sex in trail-running races as may occur in road running races. However, the previous studies included races of >100 km. Therefore, we aimed to verify the influence of performance level and sex on pacing in the last four (2017, 2018, 2019, and 2021) editions of a shorter (56.3 km) ultra-trail running race (i.e., Orsières–Champex–Chamonix; OCC^®^) that maintained the same race profile. The mean finishing time for the 5656 participants was 10 h 20 min 33 s ± 2 h 01 min 19 s. Pacing variability (CV%) was higher in high-level participants, thus showing a greater ability to adapt their pace to the race profile than low-level runners. Males also had a higher pacing variability than females although the effect sizes were small. Based on the current findings, we may recommend for non-elite OCC^®^ participants to adapt their pace to the race profile with a slower pace during uphills and a faster pace during downhills. Further studies including participants’ experience are necessary to confirm the effectiveness of this suggestion in trail-running races of variable distances.

## 1. Introduction

Trail-running is a pedestrian competition that takes place in natural environments (e.g., mountains, forests, or deserts) and is characterized by high elevation gains, variable terrain, and a maximum of 20% paved road [1]. The popularity of this sport has grown over the past few years, thus leading to an increase in the number of participants, competitions, and race formats [2,3]. This could be partly explained by the accessibility of ultra-trails (i.e., longer than 42.195 m) for non-professional runners, despite the high fitness levels required due to their physical and psychological demands and the health risks associated with this sport (e.g., physical risks such as dehydration or hypothermia caused by exposure to the weather) [3]. One of the most internationally recognized events in trail-running is Ultra-Trail *du Mont Blanc* (UTMB^®^), a franchise that organizes races that range from 15 to 300 km in distance and 1300–25,000 m in elevation gains [4].

The number of studies describing the factors related to trail-running performance has significantly increased during recent years [3]. Specifically, the distribution of speed over distance, i.e., “pacing”, which has been widely studied in other endurance sports [5], has also been analyzed in recent trail-running studies [3,6,7,8]. A positive pacing (i.e., running speed gradually decreases throughout the race) has been observed in distances from 45 to 161 km, in agreement with what has been observed in road running [8,9]. However, there is no consensus on other aspects of pacing, such as pacing variability, which can be easily computed with the coefficient of variation (CV%) of speed in the different race segments [8]. Thus, CV% describes how the speed varies in each segment with respect to the mean speed of the race. Some studies indicate that a lower pacing variability would be associated with a better performance [3,6,10], as previously observed in road marathons [11,12,13]. On the other hand, other authors found no relationship between the variability of pacing and performance in ultra-marathon races from 161 to 173 km [7,8]. In fact, one of these studies observed that the fastest runners were those who had a greater magnitude of speed loss (i.e., the slope of the linear regression of speed over the race); therefore, more studies assessing this parameter are warranted [7].

Meanwhile, these previous studies analyzed ultra-trails of more than 100 km [3,6,7,8]; therefore, the fact that participants had to run during the night, in addition to the long recovery periods between races, could have affected the pacing variability more than the performance level itself [8]. Therefore, it could be hypothesized that pacing variability depends on race distance, since in road running it has been observed that it is greater in a marathon when compared to a half marathon [14]. Further, it may be also speculated that pacing variability depends on sex. Although a recent study did not find differences between males and females during a 107 km ultra-trail [10], another study that examined differences in pacing between sexes during ~42.2 km marathons observed that speed variability was greater in males [13]. The same differences in pacing have also been observed in shorter races that ranged from one mile [15] to 10 km [16]. In addition, some studies have observed that females spend less time at the time stations, which could contribute to a better pacing strategy [10]. Therefore, it could be suggested that shorter trail-running races may exhibit differences between sexes that have not been previously observed in longer ultra-trail races.

Thus, the aim of this study was to determine the effect of performance level and sex on pacing in a shorter ultra-trail than those previously study. Following previous literature, we hypothesized that better performances and females would be associated with lower pacing variability.

## 2. Materials and Methods

### 2.1. Participants

**Figure 1:**
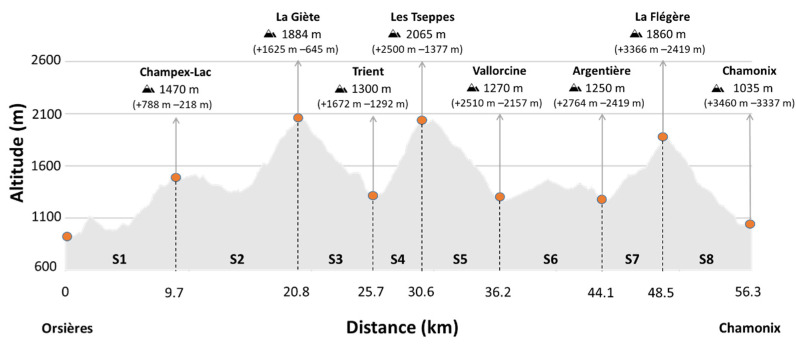
Profile, checkpoint altitude, cumulative elevation gain (+), and elevation loss (−) of the OCC^®^ 2017, 2018, 2019, and 2021 editions. S1–S8 = race segments. The horizontal axis represents cumulative distance, and the vertical axis represents altitude. Data obtained from the OCC^®^ organization.

The 5776 participants of the 2017, 2018, 2019, and 2021 editions of the *Orsières–Champex–Chamonix* (OCC^®^) ultra-trail were initially considered, of which 5655 participants (97.9%) were finally selected for the study after excluding those who did not finish the race or did not record any of the split times. The number of participants for each edition and the mean finishing times are shown in Table 1 (77.5% males and 22.5% females) (see results section). Data were retrieved from the OCC^®^ webpage [4]. This UTMB^®^ race was selected because it had less than 100 km (56.3 km and a time limit of 14 h 30 min), and a large number of high-level participants (1300–1500 participants that classified for this race by finishing other high-level ultra-trails). The course (Figure 1) did not change from 2017 to 2021. It is also important to note that the race was not held in 2020 due to the COVID-19 pandemic.

### 2.2. Race Characteristics

The first edition of the OCC^®^ took place in 2014 and was the shortest race of the UTMB^®^ until 2021. Its 56.3 km long course with 3460 m of elevation gain started in *Orsières* (Switzerland) and ended in *Chamonix* (France) and consists of 8 race segments of variable characteristics (Figure 1). The time it took each participant to complete each race segment was recorded. In the 2021 edition, the *Vallorcine* aid station time was available as well. The race is a semi-autonomous event, which means that the participants must always carry the mandatory gear (e.g., fluids, food, clothes) with them and should be self-sufficient in the whole itinerary over the six aid stations. The weather conditions (average temperature, relative humidity, and precipitation) on the day of the race of the four analyzed editions were as follows: 14.2 °C, 95%, and 16.3 mm for 2017, 16.3 °C, 76%, and 0 mm for 2018, 18.5 °C, 78%, and 1.8 mm for 2019, and 17.1 °C, 71%, and 0 mm for 2021 [17].

### 2.3. Statistical Analysis

The relative pace in each segment (%) was individually calculated for each participant based on the mean speed throughout the race (segment speed × 100/race speed). Segment speed was obtained from the participant’s time on that section and the segment distance (segment distance/participant’s segment time), and race speed by dividing the race distance by the participant’s finishing time. Pacing variability was also calculated as the CV% of relative pace. The results are expressed as mean ± SD. The analyzed participants (*n* = 5655) were stratified into four quartiles (i.e., Q1, Q2, Q3, and Q4) according to their finishing times in each edition by sex. The SPSS+ statistical software was used (v.26.0, IBM Corp., Armonk, NY, USA). The Shapiro–Wilk test was applied to ensure a Gaussian distribution of all variables. Multivariable analysis of variance (MANOVA) was used to analyze the effect of performance level (i.e., Q1, Q2, Q3, and Q4) and sex (i.e., male and female) on pacing variability, using race edition (i.e., 2017, 2018, 2019, and 2021) as a covariate. The partial eta squared (p*η*^2^) was subsequently calculated to as a measure of effect size. Partial eta squared values were classified as small (0.01–0.059), moderate (0.06–0.137), and large (>0.137) [18]. A Newman–Keuls post hoc analysis was used to establish statistical differences between means. Values of *p* < 0.05 were considered statistically significant.

## 3. Results

Table 1 shows that the mean finishing time for the 5656 participants was 10 h 20 min 33 s ± 2 h 01 min 19 s (37,233 ± 7279 s), with small significant differences between males and females (F = 166.5, *p* < 0.001, p*η*^2^ = 0.029). The race edition (2017, 2018, 2019, and 2021) had a small, significant effect on total race time (F = 4.0, *p* = 0.008, p*η*^2^ = 0.002), because the 2021 edition was faster than the previous three editions. When analyzed by sex, this effect was small in males (F = 2.7, *p* = 0.047, p*η*^2^ = 0.002) and in females (F = 6.0, *p* < 0.001, p*η*^2^ = 0.014), with no significant differences between race editions.

**Figure 2:**
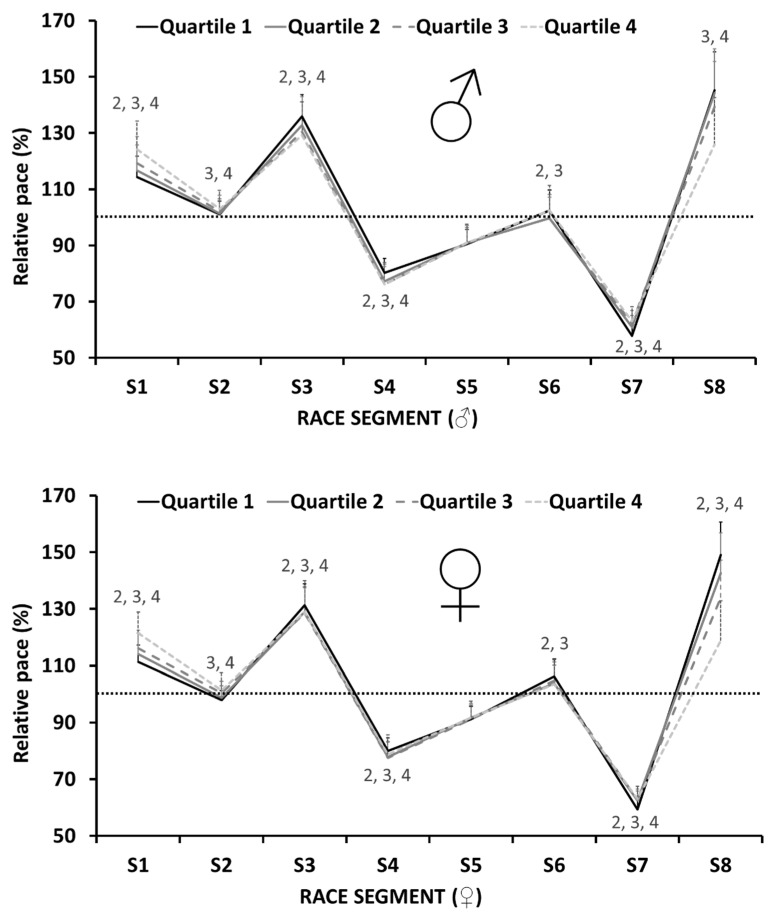
Relative pace (%) in the different race segments (S1–S8) of males (♂, *n* = 4380) and females (♀, *n* = 1276) who participated in the 2017, 2018, 2019, and 2021 editions of the OCC^®^, according to their performance levels. The numbers represent significant differences between Q1 and quartiles Q2, Q3, and Q4 (i.e., Q1 to Q2, Q1 to Q3, and Q1 to Q4).

Table 2 shows the relative pace of participants classified by performance level and sex, as well as the effect of these variables and their interaction (level × sex). Figure 2 shows significant differences in males and females between Q1 and quartiles Q2, Q3, and Q4. Overall, high-level runners had a lower relative pace in the 1st, 2nd, and 7th race segments (F = 256.3, *p* < 0.001, p*η*^2^ = 0.120; F = 50.6, *p* < 0.001, p*η*^2^ = 0.026; F = 223.3, *p* < 0.001, p*η*^2^ = 0.106, respectively), and a higher pace in the 3rd, 6th, and 8th segments (F = 41.4, *p* < 0.001, p*η*^2^ = 0.021; F = 37.2, *p* < 0.001, p*η*^2^ = 0.019; F = 526.2, *p* < 0.001, p*η*^2^ = 0.218, respectively). In addition, females had a slower pace in the first three segments (F = 102.8, *p* < 0.001, p*η*^2^ = 0.018; F = 184.2, *p* < 0.001, p*η*^2^ = 0.032; F = 67.5, *p* < 0.001, p*η*^2^ = 0.012, respectively) and a higher pace in the 6th segment (F = 305.6, *p* < 0.001, p*η*^2^ = 0.051) when compared to males, without differences in the other segments. A combined effect between level × sex was observed in the 8th segment, with larger differences between performance levels in females than in males (F = 25.0, *p* < 0.001, p*η*^2^ = 0.013).

**Figure 3:**
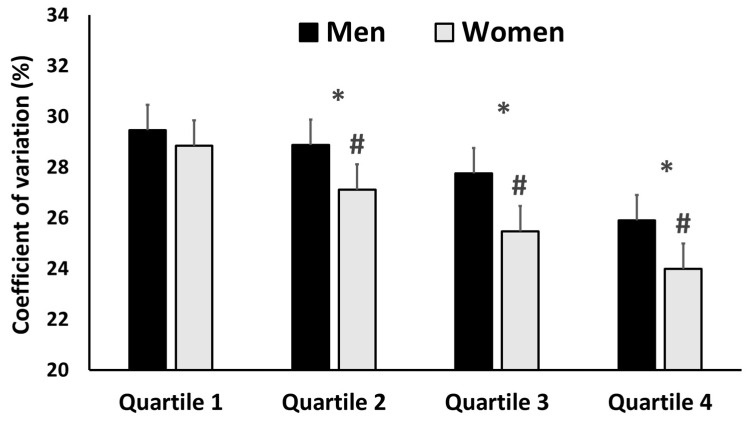
Coefficient of variation of the segment speeds for males (♂, *n* = 4380) and females (♀, *n* = 1276) who participated in the 2017, 2018, 2019, and 2021 editions of the OCC^®^, according to their performance levels (Quartile 1–Quartile 4). * Significant differences with the next quartile (i.e., Q1 to Q2, Q2 to Q3, and Q3 to Q4); # significant differences between males and females.

Figure 3 shows that pacing variability was higher in high-level participants (F = 209.2; *p* < 0.001, p*η*^2^ = 0.100). Males also had a higher pacing variability than females (F = 171.8, *p* < 0.001, p*η*^2^ = 0.030); however, no level × sex interaction was observed (F = 8.4, *p* < 0.001, p*η*^2^ = 0.004).

In the 2021 edition (see Table 3), high-level runners spent less time (F = 98.5, *p* < 0.001, p*η*^2^ = 0.182) and a lower percentage of their finishing time in the aid station (F = 48.5, *p* < 0.001, p*η*^2^ = 0.198) compared with those of a lower level. Likewise, females spent less time (F = 34.6, *p* < 0.001, p*η*^2^ = 0.025) and a lower percentage of their finishing time at aid stations than males (F = 46.6, *p* < 0.001, p*η*^2^ = 0.034), with but no level × sex interaction observed (time: F = 2.4, *p* = 0.066, p*η*^2^ = 0.005; finishing time percentage: F = 1.1, *p* = 0.358, p*η*^2^ = 0.002).

## 4. Discussion

The novel finding of the current study is that pacing during a short ultra-trail (OCC^®^, 56.3 km) differs between runners of different levels and also by sex. Thus, high-level runners exhibited a higher pacing variability, which would mean that they may have a better adaptation to the profile, changes in altitude, and segments of the race, in order to maintain a steady effort. Likewise, females’ pacing variability was lower than that of males, although this difference presented small effect sizes and could be related to the existing differences in strength between males and females.

The greater pacing variability of high-level runners (see Figure 3) is in disagreement with the results of previous studies of longer races (i.e., >100 km) that reported opposite results [3,10] or did not find any differences between performance levels [7,8]. It is difficult to establish a connection between pacing variability and ultra-trail performance, as the profile of the race directly influences the mean speed, which has been the main variable used to analyze pacing. However, it might be suggested that this is due to the greater capacity of the high-level runners to adapt their effort to the profile (i.e., uphills and downhills) and race segment (i.e., early, or last part of the race), as previous studies have stated [19]. In this regard, it is also important to note that the OCC^®^ is significantly shorter (56.3 km) than the ultra-trails that had been previously analyzed (i.e., >100 km).

It has been hypothesized that high-level athletes have a greater teleoanticipation [20], thus subconsciously adopting an optimal pacing strategy. In fact, during most of the uphill segments (i.e., S1, S2, and S7), particularly during the first and last climbs (i.e., S1 and S7, respectively), high-level runners reduced their speed more than those of a lower level, while the opposite was observed during most of the downhill segments (i.e., S3 and S8) (see Table 2). It is widely known that metabolic expenditure increases exponentially during uphill running but does not decrease similarly during downhills [21]. It is also known that trail runners who have a higher pace variation as a function of the profile of the race exhibit more constant levels of oxygen consumption [22]. Therefore, it could be suggested that high-level runners present a better adaptation to the profile of the race, thus optimizing their metabolic expenditure. This has not been observed in longer ultra-trails, probably because of the greater influence of other factors such as running during nights or spending more time in the aid stations [8]. However, our results are in line with what was observed in road running marathons with minimal elevation changes, where high-level runners have less pacing variability with the aim of maintaining their metabolic expenditure as stable as possible [11,12,13]. The lower variability of these previous studies on road was suggested to be influenced by the participants’ marathon experience (i.e., years of experience, prior marathons, prior races at all distances, and fastest previous marathon) [13]. Unfortunately, in our study we did not have access to these data. Consequently, a recommendation for non-elite OCC^®^ participants would be to adapt their pace to the race profile with a slower pace during uphills and a faster pace during downhills [19]. Further studies considering the participants’ experience are warranted to confirm the validity of this recommendation.

The lower pacing variability with small effect sizes observed in females when compared to males (see Figure 3) is somewhat similar to that observed in races with distances between a mile and a marathon [13,15,16]. As previously explained, a lower pacing variability in trail-running would have a higher impact on metabolic expenditure. In this sense, the only study that analyzed sex differences in ultra-trail races (107 km) did not find any differences between males and females [10]. Noteworthily, we may suggest that there is not enough evidence to affirm that pace regulation is clearly different according to sex. The differences in pacing variability (see Figure 3) and relative pace (see Table 2) were very small and could be justified by the physiological and psychological differences between males and females [13,15,16]. Therefore, a possible explanation for these pacing variability differences may be that males, because of their greater lean mass and thus relative strength, are potentially able to run faster during uphill sections [3]. Furthermore, sex differences in pacing variability were smaller as the level of performance increased (see Figure 3), which is in line with previous studies in marathon [13]. When looking at pacing profiles, males and females exhibited a similar relative pace (see Figure 2). Meanwhile, the differences observed in the 6th race segment between males and females could be explained by differences in the time spent at the aid station (see Table 3), as this time was included in this race segment, except for the 2021 edition.

Interestingly, high-level participants spent less time at the aid station (see Table 3) than low-level participants (large effect sizes), both in absolute and relative terms. Similarly, females spent less time compared to males but with small effect sizes. Although it was not one of the main aims of this work because aid station time was only recorded in the 2021 edition, it is noteworthy that previous studies that analyzed longer ultra-trails observed a similar association between performance and the time spent at the aid station [7,8,10]. These studies suggested that the lower pacing variability may be associated with a higher performance level. However, we may suggest that this result is not related to pacing variability, as it could be expected that high-level runners have better nutrient intake strategies, higher fitness levels, or even a better teleoanticipation, which could induce them to spend less time at the aid stations. Regarding the differences according to sex, our results are in line with those of a recent study that found differences in the time spent in the aid stations that were located before the 84th km of a longer race [10]. As we were only able to retrieve aid station time data from one edition, further research is needed to better explore this issue.

The main limitation of this study is that we were not able to obtain data of the participants’ running experience as this information was not recorded by the OCC^®^ organization. However, classifying the participants by quartiles allowed us to establish an association between performance level and pacing variability. Even though the 2021 edition was faster than the previous editions, the fact that we included this covariate in the statistical analysis means that the found differences are not biased by this edition. Additionally, the faster mean times of the 2021 edition may be explained by the lower percentage of female participation (see Table 1, 19.8% vs. 22.3–24.9%, respectively), as they have slower finishing times. Although speculative, the small performance improvement in the last race edition may also be explained by a potential selection bias after the pandemics. Thus, it may be suggested that the best runners decided to participate in the 2021 edition after the 2020 pandemics. This may also be the reason behind the reduction in the number of participants (i.e., 1340 participants in 2021 vs. 1414 participants in 2019). In addition, previous experience from the past editions may help participants to improve their pacing tactics, as the same race profile was used during these five editions. Another limitation of the present study was that we calculated pacing variability as the coefficient of variation (CV%), following previous studies on this topic [3,10]. The standard deviation was used, instead of the weighted standard deviation, which may be considered in future studies to verify whether the differences in the duration of the segments have some potential influence on pacing variability.

## 5. Conclusions

Variable pacing was found to be associated with performance in the OCC^®^ ultra-trail race (56.3 km), with high-level runners exhibiting a higher pacing variability than low-level runners. The small differences observed between sexes are not relevant enough to conclude that males and females clearly exhibit different pacing strategies. Based on the current results and another recent study [20], we may recommend for non-elite OCC^®^ participants to adapt their pace to the race profile with a slower pace during uphills and a faster pace during downhills. Further studies including participants’ experience are necessary to confirm the effectiveness of this suggestion in trail-running races of variable distances.

## Figures and Tables

**Table 1:** Mean ± SD of finishing time and number of participants of the 2017, 2018, 2019, and 2021 OCC^®^ ultra-trail.

Year	Overall (♂ + ♀)		Male (♂)		Female (♀)	
	*n*	Finishing Time (hh:mm:ss)	*n*	Finishing Time (hh:mm:ss)	*n*	Finishing Time (hh:mm:ss)
2017	1429	10:17:38 ± 02:02:09 *	1110 (77.7%)	10:02:06 ± 01:58:51	319 (22.3%)	11:11:43 ± 01:58:09 *
2018	1473	10:26:19 ± 02:01:39 *	1106 (75.1%)	10:13:11 ± 02:00:35	367 (24.9%)	11:05:53 ± 01:56:20 *
2019	1414	10:25:05 ± 01:58:2 8*	1089 (77.0%)	10:15:05 ± 01:57:55	325 (23.0%)	10:58:34 ± 01:54:14 *
2021	1340	10:12:35 ± 02:02:37	1075 (80.2%)	10:07:39 ± 02:00:34	265 (19.8%)	10:26:36 ± 02:08:55
Total (2017–2021)	5656 (100%)	10:20:33 ± 02:01:19	4380 (77.5%)	10:09:29 ± 01:59:33	1276 (22.5%)	10:58:34 ± 01:59:41

* Significant differences (*p* < 0.05) between each edition and the 2021 edition.

**Table 2:** Relative pace (%) in the different race segments (S1–S8) of males (♂, *n* = 4380) and females (♀, *n* = 1276) who participated in the 2017, 2018, 2019, and 2021 editions of the OCC^®^, according to their performance levels (Quartile 1–Quartile 4). Effect size of the differences according to performance level, sex, and their interaction (level × sex).

	LEVEL								SEX		Partial Eta Squared (pη2)		
Race Segment	Quartile 1		Quartile 2		Quartile 3		Quartile 4		Male (♂)	Female (♀)	Level	Sex	Level × Sex
	♂	♀	♂	♀	♂	♀	♂	♀					
S1	114.3 ± 7.5	111.3 ± 5.9	116.6 ± 9.0	114.0 ± 7.5	119.2 ± 9.6	116.1 ± 6.4	124.2 ± 10.0	121.5 ± 7.4	118.6 ± 9.8	115.7 ± 7.8	0.120	0.018	0.000
S2	101.1 ± 4.7	97.9 ± 3.4	101.4 ± 5.2	98.7 ± 4.2	102.1 ± 5.7	100.4 ± 4.2	103.0 ± 6.6	101.4 ± 6.2	101.9 ± 5.6	99.6 ± 4.8	0.026	0.032	0.003
S3	135.9 ± 7.8	131.3 ± 7.6	132.8 ± 10.2	129.0 ± 8.5	130.4 ± 10.7	128.5 ± 9.4	129.2 ± 11.8	129.0 ± 11.0	132.1 ± 10.6	129.5 ± 9.3	0.021	0.012	0.005
S4	80.3 ± 5.1	80.0 ± 4.6	77.3 ± 6.1	77.8 ± 5.5	76.2 ± 6.3	77.5 ± 5.6	76.2 ± 7.7	78.8 ± 6.9	77.5 ± 6.6	78.5 ± 5.8	0.029	0.005	0.005
S5	90.5 ± 5.2	91.2 ± 4.4	91.0 ± 5.4	91.7 ± 4.5	91.0 ± 6.0	91.2 ± 5.5	90.9 ± 6.7	91.7 ± 5.9	90.8 ± 5.9	91.4 ± 5.1	0.001	0.002	0.000
S6	102.4 ± 7.4	106.2 ± 6.2	99.7 ± 7.5	103.8 ± 6.4	99.7 ± 8.4	104.7 ± 6.6	102.4 ± 9.0	107.2 ± 8.6	101.1 ± 8.2	105.5 ± 7.1	0.019	0.051	0.001
S7	57.8 ± 3.7	59.4 ± 3.4	61.0 ± 4.0	62.5 ± 3.4	62.3 ± 4.5	62.8 ± 3.7	63.1 ± 5.1	63.1 ± 5.0	61.0 ± 4.8	62.0 ± 4.0	0.106	0.008	0.004
S8	145.3 ± 13.5	149.0 ± 11.6	144.3 ± 15.6	142.7 ± 14.1	139.4 ± 16.0	134.0 ± 13.2	125.9 ± 16.6	118.8 ± 14.0	138.7 ± 17.3	136.2 ± 17.4	0.218	0.005	0.013

Partial eta squared values (p*η*^2^) were classified as small (0.01–0.059), moderate (0.060–0.137), and large (>0.137).

**Table 3:** Time spent in the aid station by the 2021 OCC^®^ participants (in seconds and as a percentage of finishing times).

	Aid Station Time (s)			Percentage of Finishing Time (%)		
LEVEL	Overall (*n* = 1340)	Male (*n* = 1075)	Female (*n* = 265)	Overall (*n* = 1340)	Male (*n* = 1075)	Female (*n* = 265)
Quartile 1	281 ± 190	289 ± 205	247 ± 103	0.98 ± 0.68	1.04 ± 0.71	0.72 ± 0.45
Quartile 2	520 ± 276 *	541 ± 284 *	433 ± 221 *	1.54 ± 0.79 *	1.61 ± 0.82 *	1.27 ± 0.61 *
Quartile 3	717 ± 368 *	755 ± 381 *	559 ± 259 *	1.87 ± 0.96 *	1.98 ± 0.99 *	1.42 ± 0.65
Quartile 4	796 ± 387 *	827 ± 399 *	672 ± 310 *	1.82 ± 0.89 *	1.88 ± 0.92	1.57 ± 0.75
TOTAL	578 ± 373	603 ± 388	478 ± 283 #	1.55 ± 0.91	1.63 ± 0.94	1.24 ± 0.69 #

* Significant differences with the next higher quartile; # significant differences between males and females.

## Data Availability

Publicly available datasets were analyzed in this study. These data can be found here: https://utmbmontblanc.com/ (accessed on 10 December 2022).
